# Satisfaction in human-DSS interaction is interactively modulated by broad DSS representations and single interactions

**DOI:** 10.3389/frai.2026.1670298

**Published:** 2026-03-19

**Authors:** Alessia Musicò, Federico Fraboni, Sofia Morandini, Luca Pietrantoni, Maurizio Codispoti, Andrea De Cesarei

**Affiliations:** Department of Psychology, University of Bologna, Bologna, Italy

**Keywords:** agreement, artificial intelligence, consistency, DSS, human-DSS interaction, outcome, satisfaction

## Abstract

**Introduction:**

Satisfaction in interactions with Artificial Intelligence-based Decision Support Systems (DSSs) is a key determinant for their adoption and continued use. DSSs provide advice to support human decision-making and are increasingly implemented in domains such as healthcare, finance, and logistics. However, user satisfaction may depend on several factors, including the outcomes produced by the DSS (i.e., desirable or undesirable), its agreement with the user’s decision (i.e., confirmation or disagreement) and its consistency over time (i.e., correspondence between the system habitual behavior and its behavior in a single decision).

**Methods:**

In an online experiment (*N* = 101, ages 19–57, *M* = 26, SD = 8.58; 38.8% M), we simulated four DSSs that cooperated with humans in solving everyday decision-making problems, manipulating agreement, efficiency and consistency.

**Results:**

We observed that user satisfaction depended not only on the features of a single decision (i.e., whether the outcome was positive/negative, and whether it agreed or disagreed with the user), but also on the consistency between the overall DSS behavior and its behavior in individual decisions.

**Discussion:**

These findings suggest that users gradually develop broader mental representations of the DSS, and that satisfaction is influenced not only by isolated interactions but also by the consistency between the system’s behavior and user expectations. These insights have important implications for the design of DSSs aimed at maintaining user satisfaction over repeated interactions.

## Introduction

Artificial intelligence has become increasingly integrated into various aspects of our daily lives. In particular, Decision Support Systems (DSSs) are designed to help users make decisions and have been widely used across various fields. As these systems have become more capable of analyzing large amounts of data quickly and accurately, they have evolved from simple tools into digital collaborators (Madhavan and Wiegmann, 2007). Given the growing reliance on these systems, it is crucial to understand the factors that drive user satisfaction with the outcomes of decisions made using a DSS. Satisfaction is a key factor in determining whether a system is adopted and consistently used and can be understood as a positive emotional response arising from the evaluation of an experience.

Work-related satisfaction was initially conceptualized by Locke (1976) as a pleasant feeling related to one’s professional activity, and it was later extended to the consumer context (Oliver, 1980). In this domain, the influence of initial expectations and perceived actual performance has been highlighted. Overall, satisfaction can be described as a psychological state that arises from the interaction between an individual’s expectations and the actual outcome of an experience. When our expectations regarding an event, object, or relationship are met or exceeded, satisfaction increases, fostering continued use; conversely, a discrepancy between expectations and reality leads to dissatisfaction and the potential discontinuation of use (Bhattacherjee, 2001). In the context of DSSs, satisfaction is associated with the performance and outcomes that each DSS produces. This view is consistent with evidence from Gudigantala et al. (2011), which shows that users’ satisfaction with Web-based DSSs is primarily driven by perceived system effectiveness, which in turn depends on the perceived accuracy of the information provided and the cognitive effort required to use the system. However, other factors may modulate satisfaction in addition to outcomes. The agreement between the system’s recommendations and the user’s autonomous decisions may be particularly important because users are more likely to perceive a DSS as trustworthy and competent when its suggestions resonate with their own reasoning processes or similarity of value (e.g., Mehrotra et al., 2021; Narayanan et al., 2023).

It is possible that satisfaction is higher with systems that take decisions similar to the users, regardless of the objective quality of the outcome, as this could foster a sense of collaboration and mutual understanding. The *Computers Are Social Actors* (CASA) framework proposed by Nass and Moon (2000) suggests that people interact with computers and other digital systems using the same social rules they apply to human interactions. This framework provides a theoretical basis for hypothesizing that agreement between a DSS and the user may enhance satisfaction, in a way similar to social satisfaction in human interactions: when a DSS and the user agree on a decision, the system may be perceived as a collaborative partner. Such agreement can strengthen trust and increase acceptance of the DSS’s recommendations, regardless of the actual outcomes of the suggested decision. Studies exploring the link between decision agreement and trust in DSS indicate that the alignment between user choices and system recommendations modulates perception of the system’s usefulness (Grgić-Hlača et al., 2022). Thus, human–DSS agreement is expected to modulate user satisfaction.

Research shows that users form mental models about the technologies they use (Nass and Moon, 2000), and these representations influence individual decisions during repeated interactions. For instance, positive outcomes in a single decision may be achieved by a system that generally achieves positive outcomes (match condition), or by a system that often achieves negative outcomes (mismatch condition), and the same applies to agreement. Such consistency between DSSs representations and single decisions may further influence, at a finer level, user satisfaction.

In marketing research, many authors emphasize the importance of consistency in relation to satisfaction (see, e.g., Pulido et al., 2014), pointing out that individual interactions are less important than the cumulative experience. Cognitive theories further underscore the potential impact of consistency on satisfaction. For example, confirmation bias (Nickerson, 1998) explains how people prefer information that aligns with their beliefs, as it reduces the cognitive effort associated with processing contradictory information. Based on these insights, it is plausible that DSSs consistency enhances user satisfaction.

Most studies have primarily focused on individual system features or isolated interactions, leaving open questions about how repeated interactions influence users’ overall evaluation of a DSS. This study addresses this gap by empirically examining both single-decision factors (outcome and agreement) and the global representations users develop of a DSS through repeated interactions. Specifically, we investigate (a) whether overall DSS representations are created and influence satisfaction and (b) whether global representations of DSS interact with single decisions outcomes to shape satisfaction. By doing so, this work provides a more comprehensive understanding of the factors influencing user satisfaction with DSSs.

## Materials and methods

### Participants

The study included 101 participants, aged between 19 and 57 years (*M* = 26, *SD* = 8.58). Sample size was chosen based on a power analysis using MorePower 6.0.4 (Campbell and Thompson, 2012), aiming at identifying the required sample size for an ANOVA with repeated measure 2 × 2 × 2 × 2, with significance threshold (*α*) = 0.05, power (*β*) = 0.80, and aiming at an effect size (η^2^_p_) of 0.14, which is defined as a large effect (Cohen, 1988). The results of this analysis indicate that a sample of 42 participants was already sufficient to detect large effects. Among the recruited participants, 58.2% identified as female, 38.8% identified as male, and 3% preferred not to share their biological sex. Most participants (58.8%) held a Master’s degree. Recruitment was carried out through online social networks, using a convenience sampling approach. Specifically, the study was advertised through posts shared on social networks (Facebook, Instagram) and messaging groups (WhatsApp, Telegram), inviting users to participate voluntarily. Interested individuals accessed the study via a link that directed them to the online questionnaire. The only requirements to take part were being at least 18 years old and having a good understanding of the Italian language. No financial compensation was offered for participation.

All participants gave electronic consent prior to participation, which was entirely voluntary, and no rewards such as payments or course credits were offered. The study was approved by the University’s Research Ethics Committee and followed ethical guidelines for psychological research.

### General procedure

An online questionnaire was designed. At the beginning of the study, participants were made to believe that they would be interacting with four real DSSs in the development phase. Each DSS required participants to collaborate with the system and consisted of 10 real-life scenarios in which the participant had to make a choice. As a result, each participant completed 40 scenarios. The aim is to analyze whether users’ satisfaction with the outcomes of decisions made in collaboration with a DSS is influenced solely by the quality of the outcomes (positive or negative) or also by the degree of agreement with the user, and to investigate the role of consistency in the system’s behavior (in terms of efficiency and agreement) within a single trial compared to its overall performance. The study employed a 2 × 2 × 2 × 2 within-subjects design, including the following factors: single-decision agreement (agree vs. disagree), single-decision outcome (positive vs. negative), overall DSS agreement (high vs. low), and overall DSS performance (high vs. low).

### Real-world problem construction and procedure

**Figure 1:**
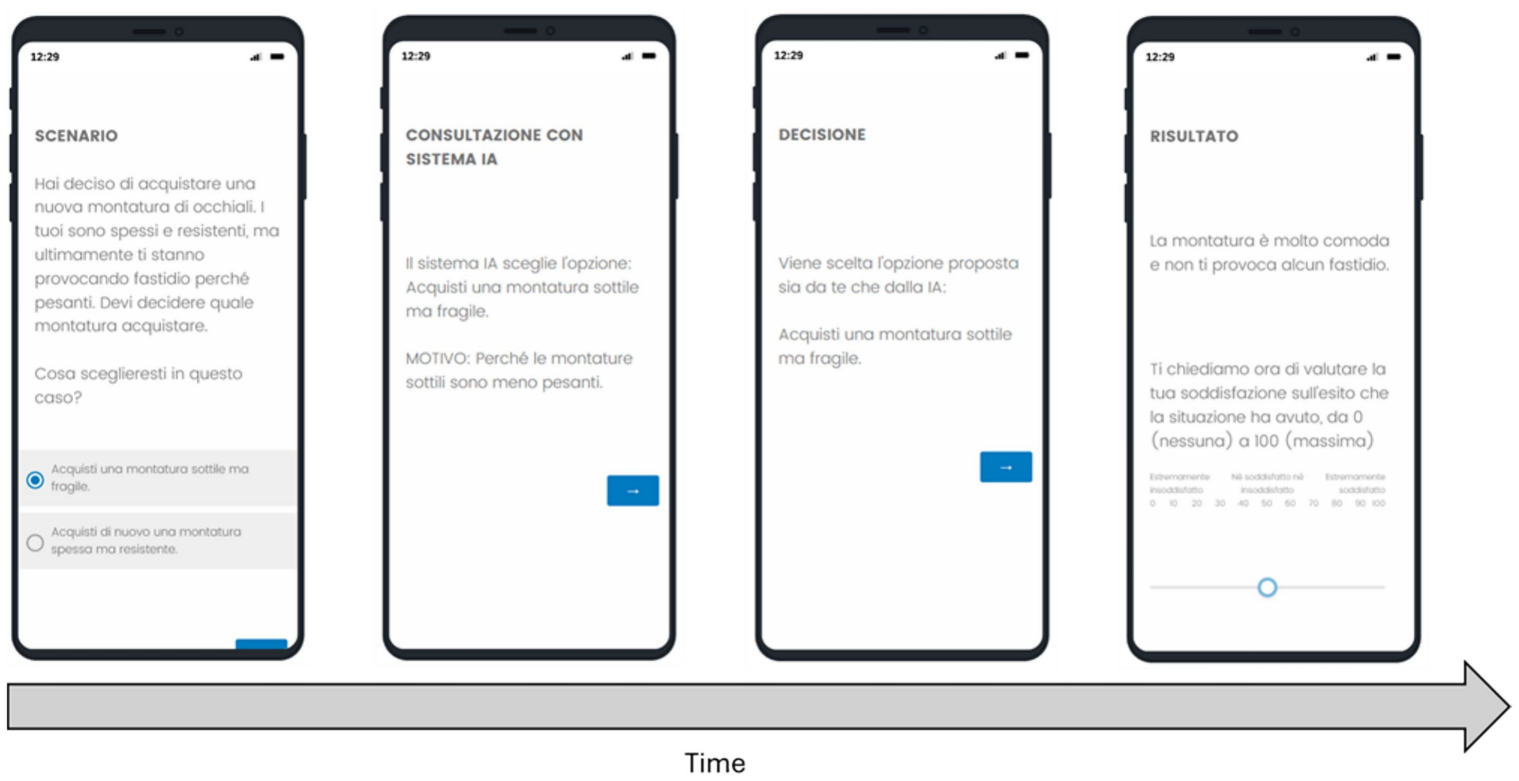
Experimental procedure for each scenario consisted of the presentation of one scenario, consultation with an AI system, decision, and evaluation of the result by the human user.

Two researchers constructed the real-world problem scenarios, and a third evaluated them. The interaction between the participant and the DSS is shown in Figure 1. In each trial, participants were confronted with a real-world problem, such as *finding the best way to*… (see Table 1). To ensure that participants’ choices covered various domains, the scenarios represented 10 different topics: clothing, food, competitions, work, prices, health, entertainment, sports, weather, and travel. Additionally, to avoid length-related effects, the scenarios were designed to be similar in size, with an average of 23 words (*SD* = 11 words). Each scenario presented two possible response options, with both positive and negative outcomes. After participants made their choice, the system presented the AI’s choice. The system then selected an action and described the outcome. The final decision could align with the user’s, the AI’s (if they disagree), or both (if they agree). Cases where the decision made is the one chosen by the user despite the AI’s disagreement are considered ‘fillers’ and are not analyzed for the purposes of this research, so the cases effectively evaluated in the analyses are 8 per DSS. Before each set of scenarios, participants were presented with a preliminary decision scenario to familiarize themselves with the procedure. After each scenario, participants were asked to rate their satisfaction with the outcome on a scale of 0 to 100, where 0 represented no satisfaction and 100 maximum satisfaction.

**Table 1:** Sample scenario with choice options and possible outcomes.

Scenario#1: ‘Today you are planning a sightseeing trip to the city of Rome. It’s a hot summer day but the weather forecast reports the likelihood of a thunderstorm. You have to decide which walking shoes to wear.’		
Choice	Positive outcome	Negative outcome
#1. Wear cool, breathable canvas shoes.	It did not rain and, despite the heat, wearing breathable shoes allowed you to continue your tour of the city with ease.	During the day the storm came, and your feet got wet, preventing you from continuing your tour of the city in comfort.
#2. You wear waterproof, non-breathable shoes.	It rained but your feet stayed dry, allowing you to continue your tour of the city.	The thunderstorm did not arrive during the day and, due to the high temperatures, the excessive heat on your feet prevented you from comfortably continuing the city tour.

### DSS

The real-world problems were organized into four DSSs, with each system consisting of 10 scenarios. All participants completed all scenarios but the order in which the four DSSs were presented was balanced among the participants. Out of the total 10 scenarios, 2 served as fillers in which DSS and humans disagreed, and the action chosen by the human user was taken. In all other cases, the action which was chosen by either the DSS alone, or by the DSS and human participant, was taken.

### Experimental manipulation

For each scenario (single-decision level), agreement with the human user and outcome were systematically manipulated. In scenarios with agreement factor set to “agree,” the decision taken by the human and the DSS was the same, while in the “disagree” condition they differed. In scenarios with the outcome factor set to “positive,” the action taken (whether it was taken by the human, AI, or both) led to a desirable outcome, while in the “negative” condition it led to an undesirable outcome.

At the overall level, the percentage of times the DSS agreed with the participant was manipulated across scenarios (i.e., the number of scenarios within a DSS in which the AI agreed with the participant was defined depending on the experimental condition). In DSSs with high agreement, the AI made decisions consistent with the participant’s choices in 6 out of 10 cases, in those with low agreement, in only 4 out of 10 cases. For DSSs that produced more positive outcomes, positive outcomes occurred in 6 out of 10 scenarios, while DSSs with more negative outcomes had only 4 out of 10 positive outcomes. As a result, DSSs could be overall agreeing, disagreeing, well-performing or bad-performing.

**Figure 2:**
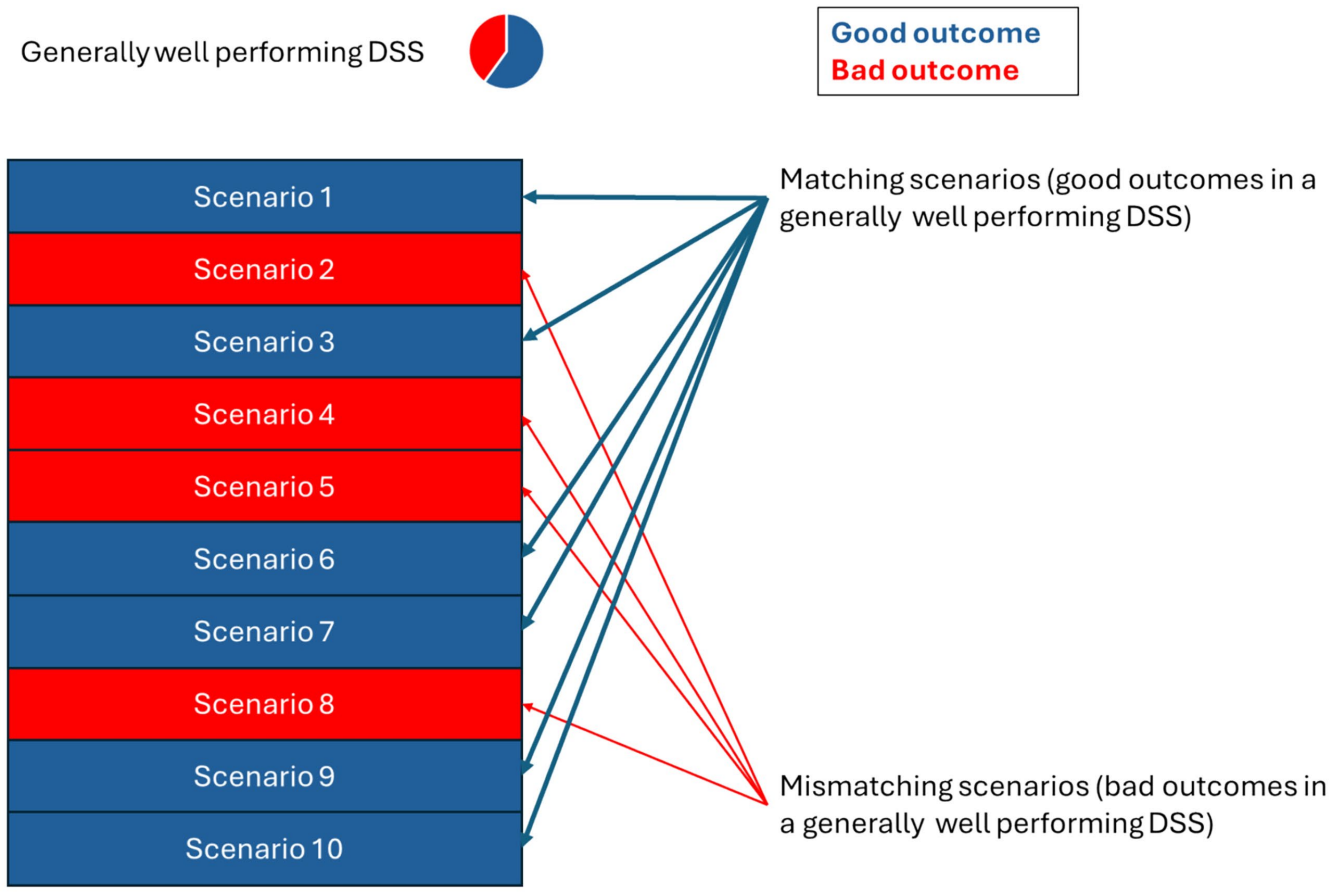
Example of matching and mismatching scenarios.

When the single-decision and overall level were consistent (e.g., an agreeing decision within a generally agreeing DSS), the scenario was coded as matching (Figure 2), while if they were not consistent the scenario was coded as mismatching. Trials in which AI and humans disagreed, and in which the decision of the participants was taken, were considered as fillers and not analyzed; therefore, the distribution of agree/disagree condition out of the remaining eight non-filler trials was 6/2, while the distribution of good/bad trials over the remaining eight non-filler trials was 5/3.

### Statistical analyses

To investigate how the characteristics of the DSS and the characteristics of individual trials affected participants’ satisfaction, a repeated-measures variance analysis (ANOVA) was used. The analysis considered both outcomes (which could be positive or negative) and levels of agreement (agreement or disagreement), distinguishing between overall dimensions and those related to single decisions. Specifically, at the overall level two factors were considered: the average of the outcomes provided by the DSS (overall outcome: overall well performing vs. overall poorly performing) and the average agreement between the participant and the DSS (overall Agreement: overall agreeing vs. overall disagreeing). At the single decision level, the focus was on the specific outcome in each single trial (single-decision outcome) and on the agreement between the participant’s choice and the DSS’s suggestion in each single trial (single-decision agreement). Where necessary, *post hoc* comparisons were carried out to investigate differences between experimental conditions, and the partial eta squared is reported as a measure of effect size.

## Results

Concerning single decisions, we observed that single-decision outcome had a statistically significant effect on participants’ satisfaction [*F*(1,100) = 292.02, *p* < 0.001, *η^2^_p_* = 0.74, mean difference = 42.51; for comparison of descriptive statistics; see Table 2], with higher satisfaction for positive compared with negative outcomes. Moreover, human-DSS agreement in single decisions significantly modulated satisfaction [*F*(1,100) = 166.46, *p* < 0.001, *η^2^_p_* = 0.625, mean difference = 20.52], with higher satisfaction when the DSS’s decision aligned with the participant’s own choice during a single decision.

**Table 2:** DSS and single decision’s outcome and agreement descriptive statistics.

Factor	Level	Mean	SD
DSS overall outcome	Mostly positive	59.32	9.44
	Mostly negative	57.42	9.07
DSS overall agreement	Mostly agreeing	57.73	9.39
	Mostly disagreeing	59.02	9.49
Single decision outcome	Positive	79.60	11.25
	Negative	37.09	18.10
Single decision agreement	Agreeing	68.63	10.77
	Disagreeing	48.11	12.38

Considering overall DSS performance through all the problem-solving session as a whole, we observed that achieving positive results had a statistically significant effect on satisfaction [*F*(1,100) = 6.075, *p* = 0.015, *η^2^_p_* = 0.057, mean difference = 1.92], with higher satisfaction for DSSs with a better, compared with worse, performance. On the other hand, overall agreement between the DSS and participants’ decisions did not significantly modulate satisfaction [*F*(1,100) = 2.316, *p* = 0.131, *η^2^_p_* = 0.023, mean difference = −1.29].

After having established the main effects, at the overall and single-trial levels, of agreement and outcome, we aimed to test whether overall and single-decision factors interact with each other in modulating satisfaction. We tested this possibility first concerning the overall and single-decision interactions of each factor (outcome and agreement), and then by examining the relationship between outcome and agreement at the overall and single-decision level.

### Relationship between overall and single-decision’s level

#### Outcome

**Figure 3:**
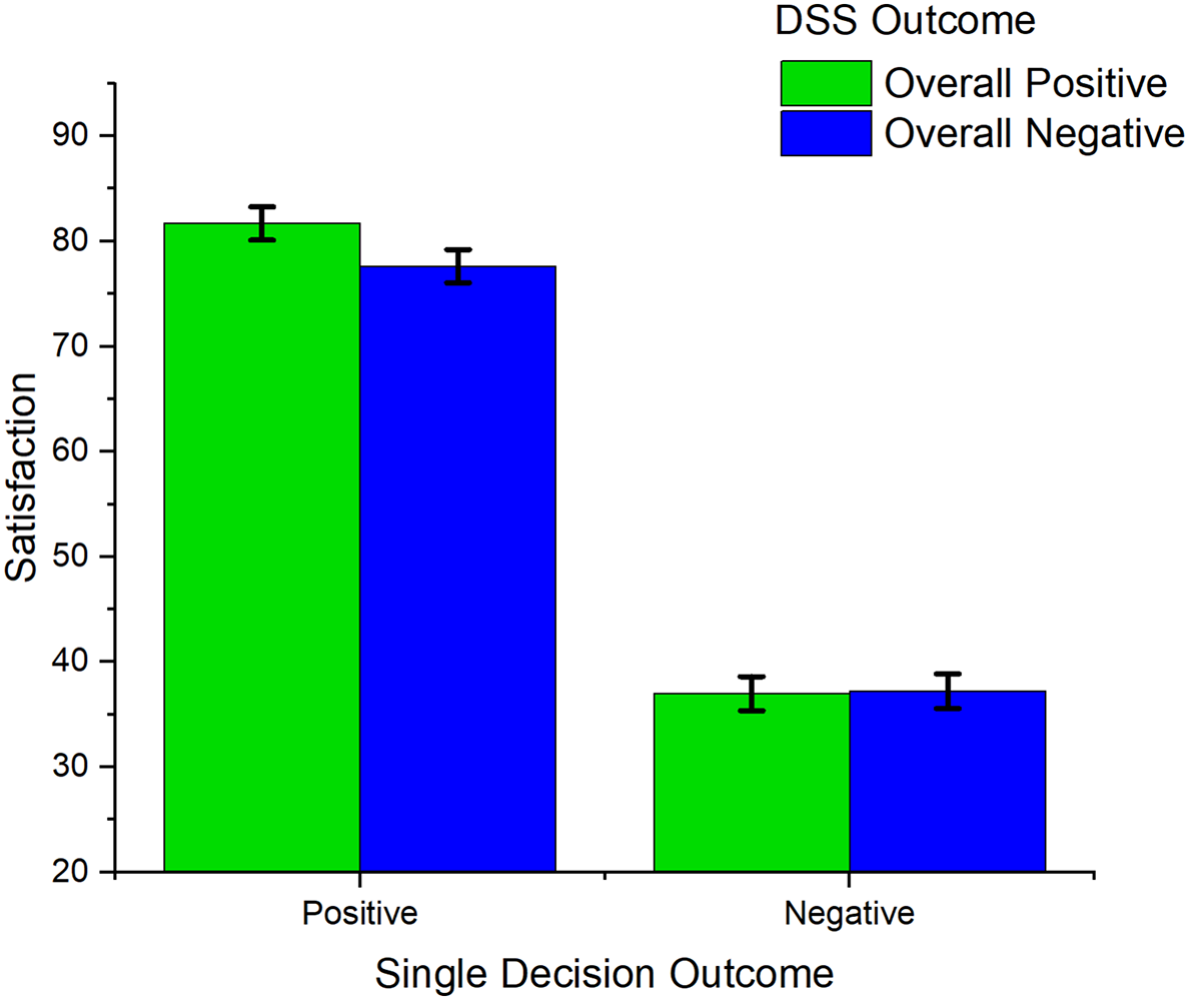
Interaction between overall DSS outcome and outcome of single decision on Satisfaction. Error bars represent within-participant standard error of the mean (O’Brien and Cousineau, 2014).

The ANOVA revealed a significant interaction between overall outcome and outcome of single decisions on satisfaction [*F*(1,100) = 5.04, *p* = 0.027, *η^2^_p_* = 0.048] (see Figure 3). When the outcome of a specific decision was positive, satisfaction was higher in overall well performing DSSs, compared to poorly performing DSSs [*F* (1, 100) = 13.18, *p* < 0.001, *η^2^_p_* = 0.116, mean difference = 4.05]. No significant difference was observed between overall well performing and poorly performing DSSs when the outcome of a single decision was negative [*F*(1, 100) = 0.006, *p* = 0.93, *η^2^_p_* < 0.001, mean difference = 0.102]. Satisfaction was significantly higher in decisions with a positive outcome compared to those with a negative outcome, both for highly efficient DSSs [*F*(1, 100) = 283.7, *p* < 0.001, *η^2^_p_* = 0.73, mean difference = 44.48] and poorly performing DSSs [*F*(1, 100) = 130.5, *p* < 0.001, *η^2^_p_* = 0.69, mean difference = 40.33].

#### Agreement

**Figure 4:**
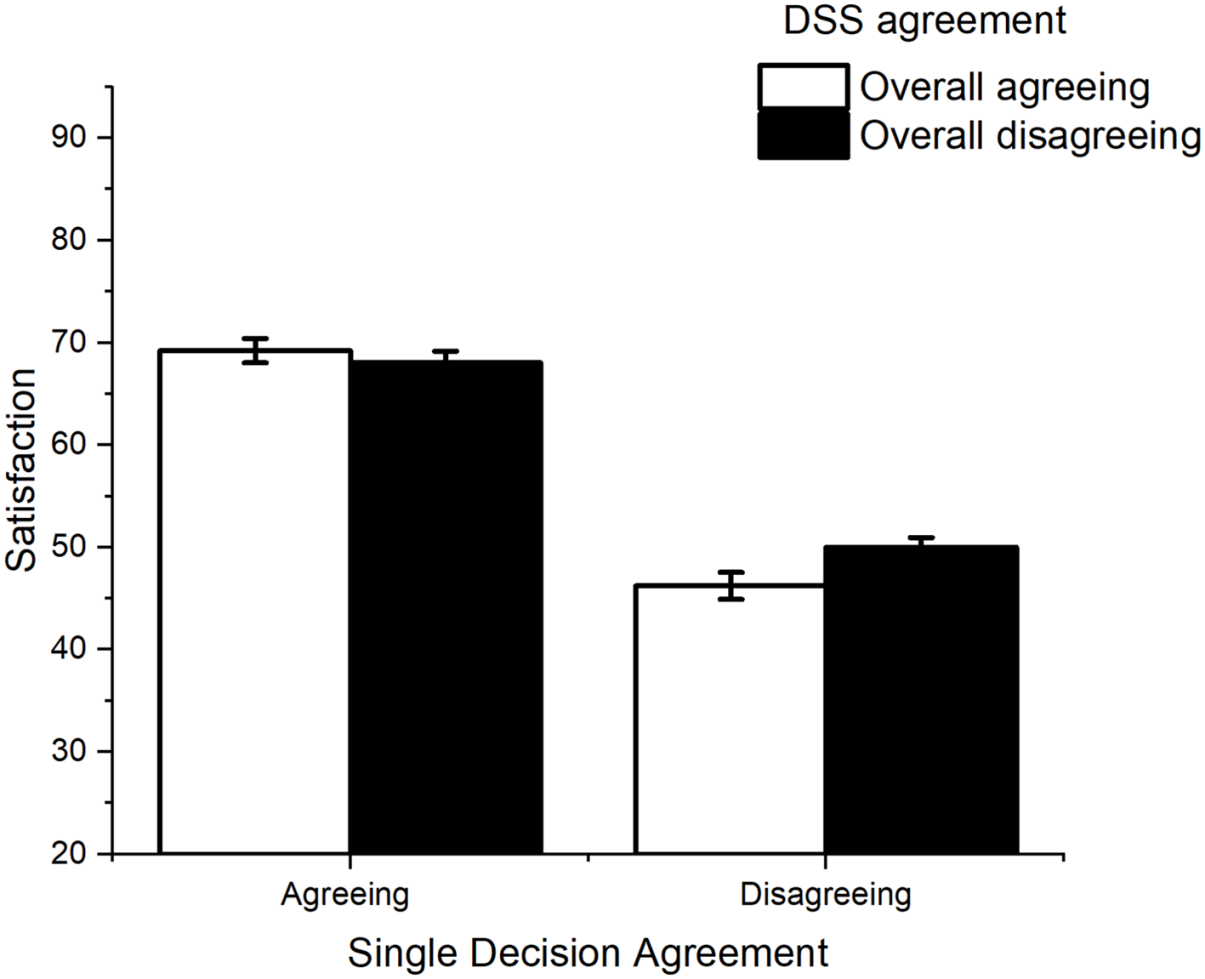
Interaction between DSS overall agreement and agreement of single decision on Satisfaction. Error bars represent within-participant standard error of the mean (O’Brien and Cousineau, 2014).

A significant interaction was found between the DSS’s overall agreement and the single decision’s agreement in influencing satisfaction with the outcome [*F*(1,100) = 8.49, *p* = 0.004, *η^2^_p_* = 0.078; see Figure 4]. When there was disagreement between the human participant and the DSS in single decisions, satisfaction was significantly higher if participants were interacting with a generally disagreeing DSS than if participants were interacting with a generally agreeing DSS [*F*(1, 100) = 9.34, *p* = 0.003, *η^2^_p_* = 0.085, mean difference = 3.75]. When human participants and DSSs agreed on single decisions, the level of satisfaction did not differ significantly depending on the overall agreement level [*F*(1, 100) = 0.77, *p* = 0.382, *η^2^_p_* = 0.008, mean difference = 1.17]. Satisfaction was significantly higher in agreeing single decisions compared with disagreeing ones, both for overall high agreement DSSs [*F*(1, 100) = 134.16, *p* < 0.001, *η^2^_p_* = 0.571, mean difference = 22.85] and for overall low agreement DSSs [*F*(1, 100) = 132.3, *p* < 0.001, *η^2^_p_* = 0.567, mean difference = 18.15].

### Relationship between outcome and agreement

**Figure 5:**
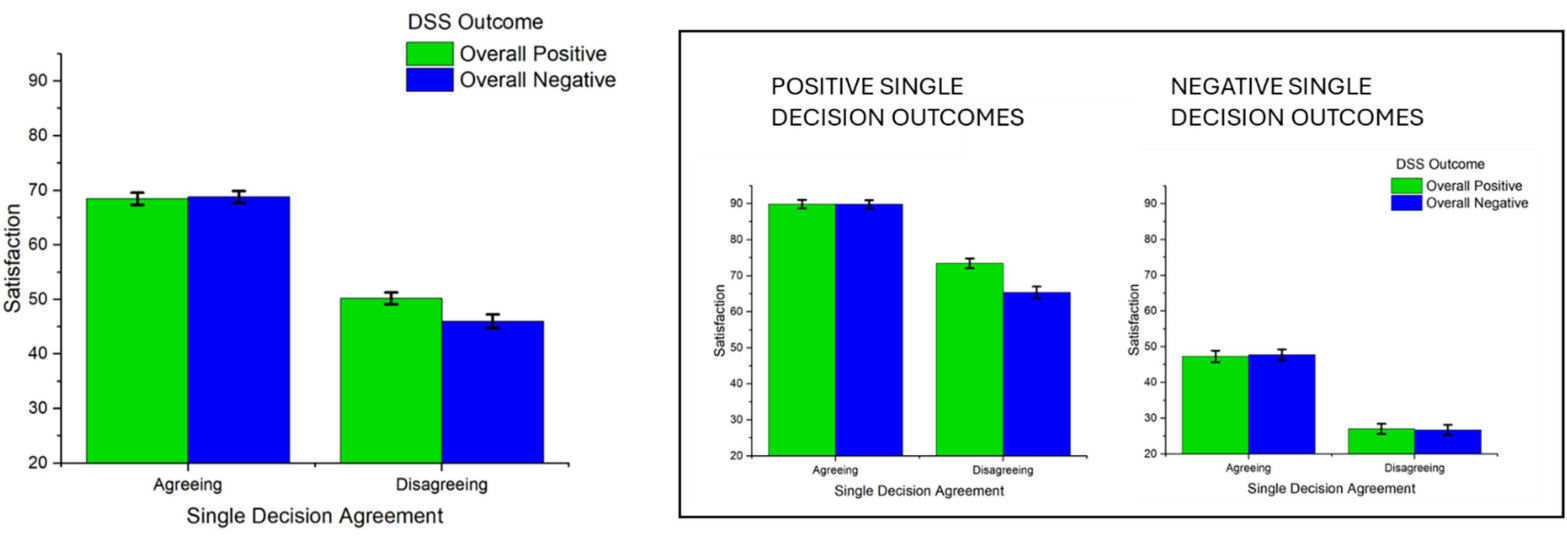
Interaction between overall DSS outcome and single-decision agreement on satisfaction, with inset: interaction between DSS average outcome, single-decision outcome and single-decision agreement. Error bars represent within-participant standard error of the mean (O’Brien and Cousineau, 2014).

A significant interaction was observed between overall outcome and single decision agreement [*F*(1,100) = 6.89, *p* = 0.010, *η^2^_p_* = 0.064; see Figure 5]. In particular, when participants were asked to rate their satisfaction of a decision not in agreement with them, they were significantly more satisfied if they were in a generally well-performing DSS (with a high percentage of positive outcomes) than in a poorly-performing DSS [*F*(1,100) = 11.70, *p* < 0.001, *η^2^_p_* = 0.104, mean difference = 4.2]. In contrast, no differences were observed for agreeing single decisions depending on whether they occurred within a well-performing or a poorly-performing DSS [*F*(1,100) = 0.038, *p* = 0.845, *η^2^_p_* < 0.001, mean difference = 0.35]. Satisfaction was significantly higher in decisions in agreement with the participant compared to those in disagreement, both for highly efficient DSSs [*F* (1, 100) = 112.9, *p* < 0.001, *η^2^_p_* = 0.528, mean difference = 18.28] and for poorly performing DSSs [*F* (1, 100) = 149.4, *p* < 0.001, *η^2^_p_* = 0.597, mean difference = 22.72].

The relationship between overall outcome and single-decision agreement was further modulated by single-decision outcome [*F*(1,100) = 6.13, *p* = 0.015, *η^2^_p_* = 0.058, see Figure 5 inset]. Regarding decisions which ended up in a desirable outcome, a two-way interaction between overall outcome and single-level agreement was observed [*F*(1, 100) = 14.86, *p* < 0.001, *η^2^_p_* = 0.128]. In scenarios where decisions disagreed between human participants and DSS, higher satisfaction was observed in well-performing compared with poorly performing DSSs, [*F*(1, 100) = 17.36, *p* < 0.001, *η^2^_p_* = 0.147, mean difference = 8.07]. No difference depending on overall outcome was observed for scenarios in which single decisions were in agreement with the user [*F*(1, 100) = 0.001, *p* = 0.973, *η^2^_p_* < 0.001, mean difference = 0.04]. Finally, satisfaction was generally higher in decisions in agreement than in disagreement with the participant in both high-efficiency [*F*(1, 100) = 76.22, *p* < 0.001, *η^2^_p_* = 0.430, mean difference = 16.50] and low-efficiency DSSs [*F* (1, 100) = 122.1, *p* < 0.001, *η^2^_p_* = 0.547, mean difference = 24.60]. In single decisions which ended up in an undesirable outcome, no significant two-way interaction between overall outcome and single-decision agreement was observed, [*F*(1, 100) = 0.146, *p* = 0.704, *η^2^_p_* = 0.001] and satisfaction was modulated by single-decision agreement, [*F*(1, 100) = 114.3, *p* < 0.001, *η^2^_p_* = 0.53].

**Figure 6:**
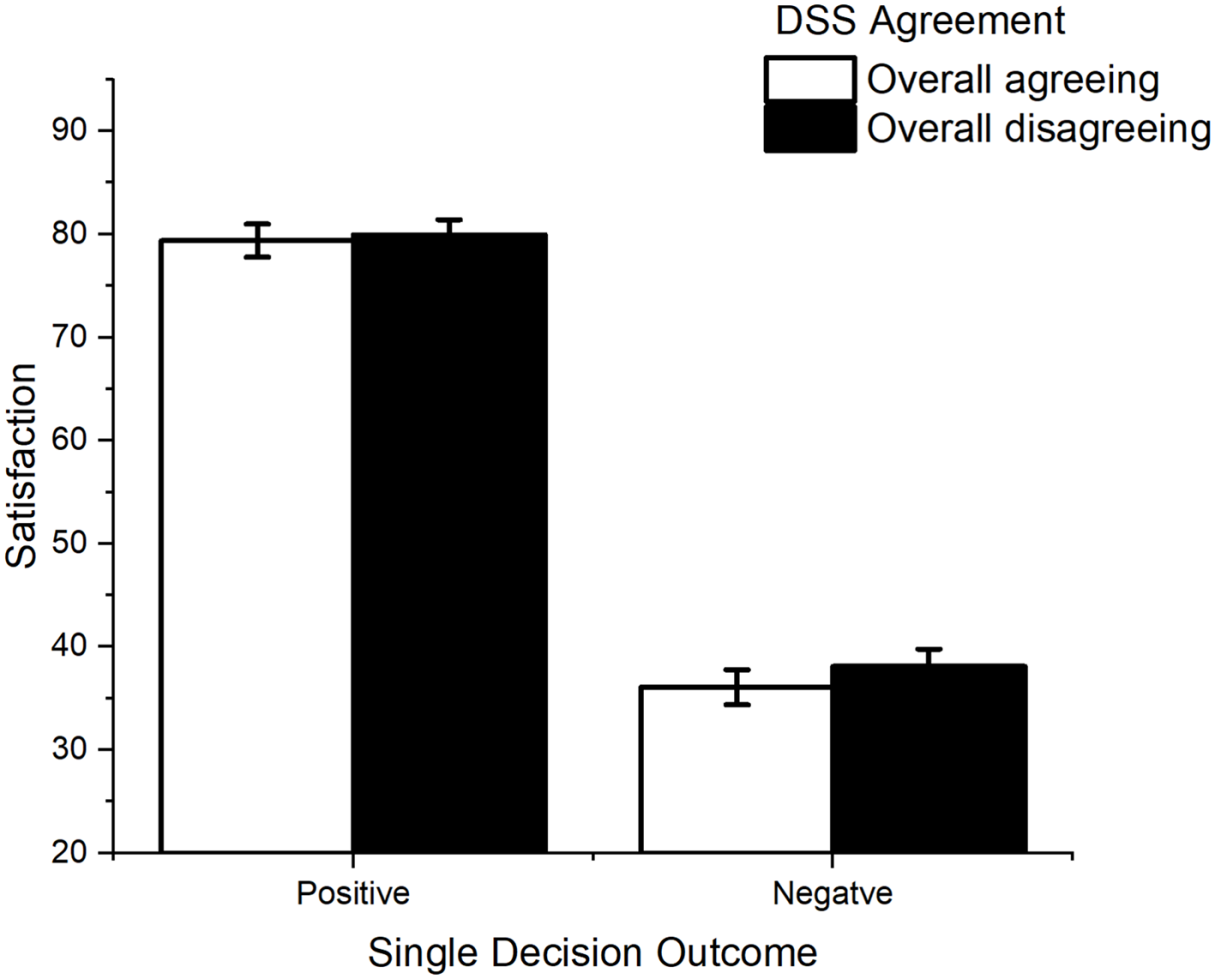
Interaction between DSS overall agreement and outcome of single decision Outcome. Error bars represent within-participant standard error of the mean (O’Brien and Cousineau, 2014).

Finally, no significant interaction between overall agreement and single-decision outcome was observed, [*F* (1, 100) = 0.65, *p* = 0.421, *η^2^_p_* = 0.006; Figure 6]. All ANOVA results are reported in Table 3.

**Table 3:** Multivariate test of main effects and interactions.

Effect	F	*p*	*η^2^_p_*
Overall outcome	6.08	0.015	0.057
Overall agreement	2.32	0.131	0.023
Single outcome	292.02	<0.001	0.745
Single agreement	166.46	<0.001	0.625
Overall outcome × Overall agreement	3.47	0.065	0.034
Overall outcome × Single outcome	5.04	0.027	0.048
Overall agreement × Single outcome	0.65	0.421	0.006
Overall outcome × Overall agreement × Single outcome	1.88	0.173	0.018
Overall outcome × Single agreement	6.89	0.010	0.064
Overall agreement × Single agreement	8.49	0.004	0.078
Overall outcome × Overall agreement × Single agreement	0.55	0.462	0.005
Single outcome × Single agreement	0.01	0.929	<0.001
Overall outcome × Single outcome × Single agreement	6.16	0.015	0.058
Overall agreement × Single outcome × Single agreement	0.40	0.527	0.004
Overall outcome × Overall agreement × Single outcome × Single agreement	3.91	0.051	0.038

## Discussion

The present study aimed to investigate how the relationship between the overall representations of a DSS and decision-level factors (i.e., outcome and agreement) influences user satisfaction. Specifically, we examined whether satisfaction is shaped solely by the characteristics of individual decisions or whether it is also influenced by broader representations of the system formed through repeated interactions. The findings suggest that user satisfaction is not only a function of isolated decision outcomes, but is systematically modulated by how single decisions align with the user’s global representation of the DSS.

Not surprisingly, both the outcome and the human-DSS agreement of single decisions modulated satisfaction. Specifically, trials with positive outcomes generated higher levels of satisfaction among participants, and the same applied to trials in which DSSs agreed with the participants’ choices. Moreover, the overall performance of the DSS also had a significant impact on satisfaction, with higher overall performance levels associated with higher satisfaction. In contrast, overall human-DSS agreement did not show a main effect on satisfaction. At first glance, this result is apparently at odds with previous studies showing that similarity between AI and humans fosters acceptance (Mehrotra et al., 2021; Narayanan et al., 2023; Grgić-Hlača et al., 2022). However, we also observed that overall agreement interacted with decision-level agreement (see Figure 4), suggesting that an overall representation of DSS’s agreement was formed. Collectively, the results suggest that individuals develop representations of the specific DSS they are interacting with, and that these attitudes - similar to what occurs in social relationships - can be modulated by overall behavior. These results are consistent with the CASA framework (Nass and Moon, 2000), which proposes that the interactions between humans and technological artifacts (i.e., computers and, in the present case, DSSs) are regulated by social rules and representations akin to those governing human interactions.

Overall representations of DSSs dynamically interacted with single-decision features to modulate satisfaction. As the social representation of the artificial partner develops, it may match or mismatch the behavior of the DSS in each single decisional instance. For instance, a negative outcome may be achieved by a system which generally achieves positive outcomes (mismatch condition), or by a system which often achieves negative outcomes (match condition). Supporting the relevance of this distinction, in the present study significant interactions between overall and single-decision factors were observed concerning both outcome and agreement. More specifically, when overall factors (outcome or agreement) matched or mismatched single-decision outcome or agreement, satisfaction was modulated accordingly. Concerning the outcome, higher satisfaction was observed in decisions with positive outcomes when overall system performance was also positive, compared to when overall system performance was negative (see Figure 3). Concerning agreement, we observed that decisions in which humans and DSSs were in disagreement led to lower satisfaction when they occurred in the context of a high-agreement DSS, i.e., when they mismatched overall agreement. In sum, these results align with previous observations that satisfaction crucially depends on the consistency of a system (Pulido et al., 2014), both at the level of outcome efficiency (where overall positive performance boosted satisfaction for positive decisions), and agreement (where decision-level disagreement led to lower satisfaction if it happened in a generally agreeing system).

An overall representation of DSS outcome and agreement was established, as evidenced by significant main effects or interactions involving these overall factors. Within the inherently collaborative nature of shared activities between humans and DSSs (Madhavan and Wiegmann, 2007), this finding suggests that outcome and agreement are critical dimensions shaping the social representation of the partner, whether human or artificial, with whom such activities are carried out. Such creation of global representation of technological artifacts we are interacting with can be associated with anthropomorphism, i.e., “the human propensity to attribute human characteristics to non-human entities” (Epley et al., 2007; Nass and Moon, 2000; Bartneck et al., 2009; Cabitza et al., in press). In this light, it can be asked which properties of a system can be anthropomorphized (Kim and Im, 2023; Epley et al., 2007; Cacioppo and Hawkley, 2009), at the functional, appearance or psychological level.

The results of this study seem to suggest that the overall outcome is a more characterizing feature of DSS representation compared with overall agreement. Concerning the relationship between outcome and agreement, we examined the reciprocal interaction of overall outcome on single-decision agreement, and viceversa of overall agreement on single-decision outcome (see Figure 5). Interestingly, we observed an asymmetric pattern of results, with overall outcome modulating the effects of single-decision agreement on satisfaction, but not viceversa. More specifically, when human and DSS decisions were in disagreement, satisfaction was higher if interacting with a generally efficient DSS, compared to a poorly performing one, and this effect emerged only in trials with a positive outcome. This could indicate that participants interpret disagreement as a sign of the system’s expertise, and can be seen as a social reasoning process: disagreement with a competent agent can be perceived as expert guidance rather than conflict. Conversely, with a low-performing DSS, the same scenario highlights inconsistency and reduces satisfaction. This interpretation aligns with Festinger’s (1957) cognitive dissonance theory, which posits that conflicting (i.e., disagreeing with the user) information can amplify dissatisfaction when it challenges beliefs about system competence.

Taken together, these patterns also have direct implications for the design of DSSs. The findings indicate that user satisfaction is shaped not only by the accuracy or agreement of individual decisions, but also by the consistency of system behavior over time. Users appear to evaluate individual recommendations relative to an inferred “system profile,” built through repeated interactions. From a design perspective, this suggests that maintaining stable and predictable behavioral patterns may support more robust user satisfaction than optimizing individual decisions in isolation.

The results further point to a trade-off between agreement and consistency. Systems that frequently disagree with users may nonetheless sustain satisfaction when they display consistently high performance, as disagreement can be interpreted as expert guidance within a context of demonstrated competence. Conversely, occasional disagreement within a generally agreeing system may generate disproportionate dissatisfaction, as it violates established expectations. This suggests that agreement may primarily support short-term satisfaction, whereas consistency contributes more strongly to the stability of satisfaction over repeated interactions.

These findings also highlight the role of interface design in managing user expectations. Rather than relying solely on users’ implicit learning of system behavior, DSS interfaces could support expectation calibration by making global system characteristics more transparent. For example, feedback summarizing recent performance trends, typical agreement rates, or variability across decisions may help users contextualize unexpected outcomes or disagreements.

Moreover, communicating uncertainty emerges as a relevant design lever. Signals such as confidence estimates, probability ranges, or brief explanations for atypical recommendations may help users reinterpret deviations from expected behavior and reduce negative expectancy violations. By aligning system feedback with actual decision patterns, DSSs may foster more coherent global representations, thereby supporting stable user satisfaction across repeated use.

Finally, when decisions were in agreement, overall DSS performance did not affect satisfaction: agreement at the single-decision level increased satisfaction, independent of the system’s overall efficiency. This pattern might suggest that users prioritize relational cues - such as agreement with the DSS - over global assessments of competence. In these situations, agreement may function as a form of social validation, buffering dissatisfaction even when overall performance is poor.

Overall, these findings indicate that designing DSSs for repeated use requires attention not only to local decision quality, but also to how system behavior unfolds over time. By shaping consistent behavioral patterns and supporting users in forming calibrated expectations, DSSs may sustain user satisfaction across repeated interactions.

### Limitations and future directions

While this study provides valuable insights into the cognitive processes underlying DSS satisfaction, several factors may limit the ecological validity and generalizability of the findings. First, the experimental scenarios were low-stakes and narrative-based, which may not have elicited the emotional or cognitive engagement typical of real-world DSS applications. This design allowed us to investigate fundamental mechanisms of satisfaction, such as agreement between user and system, consistency across decisions, and the formation of global system representations. Classically, real DSS often involve high-stakes decisions (for example in healthcare, finance, or legal contexts), with tangible consequences. However, AI tools and DSSs are increasingly used outside of high-risk scenarios, e.g., when browsing information, booking a holiday, or online shopping. It is necessary to be cautious when generalizing these results to real-world deployments, as effect magnitudes and user experiences may differ across separate domains (e.g., high-stakes contexts, online shopping, information gathering, etc.). Second, satisfaction was measured via self-report ratings; including additional behavioral measures such as reliance, trust scales, or confidence ratings could provide a more robust understanding of user responses. Third, the participant pool was predominantly young and well-educated (58.8% with Master’s degrees), which limits generalizability to broader populations. Fourth, the development of system representation across repeated trials should be better explored by systematically varying the degree of experience with a system.

Acknowledging these limitations emphasizes that findings should be interpreted within the constraints of the experimental setup, while highlighting the novelty of our methodology, which directly observes interactions between users and systems - a challenging approach rarely implemented in DSS research.

Building on these limitations, future research could expand on this work by validating the results with real AI-based DSSs. Furthermore, it may be useful to implement longitudinal projects to observe the evolution of satisfaction over days or weeks and include behavioral measures of adherence to the system’s recommendations and external satisfaction ratings. These approaches would strengthen ecological validity and provide a more comprehensive understanding of how user satisfaction develops in the actual use of DSSs.

## Conclusion

This study aimed to explore how user satisfaction is influenced by the interplay between single-decision and overall performance of DSSs. Users’ experiences were not only shaped by isolated factors, but also by the interaction between single trial outcomes and agreement and the system’s broader performance and behavior. While single-decision agreement and outcome consistently shaped satisfaction, their impact was moderated by the broader context in which they occurred. In particular, although positive outcomes generally increase satisfaction, their effect was amplified when embedded in globally efficient systems. Similarly, disagreement trials resulted in lower satisfaction when mismatching with an overall agreeing DSS. These findings suggest that to sustain high user satisfaction, DSSs must strike a balance between consistent performance and alignment with user expectations. In particular, systems that frequently disagree with users must compensate by maintaining high efficiency to mitigate dissatisfaction. This study enhances our understanding of how cognitive biases influence user satisfaction with DSSs. Gaining insight into these biases can be highly valuable for developers, enabling them to optimize user experience and foster greater satisfaction in interactions with DSSs.

## Data Availability

The raw data supporting the conclusions of this article will be made available by the authors, without undue reservation.
